# Nrf2 Is Associated with Metastasis-Related Processes in a Chemoresistant Breast Cancer Model: Insights from siRNA Modulation

**DOI:** 10.3390/ijms27104506

**Published:** 2026-05-18

**Authors:** Andrea Muñoz-Ayala, Nicolás Serafín-Higuera, Ana Gabriela Leija-Montoya, Octavio Galindo-Hernández, José Luis Vique-Sánchez, Raúl Díaz-Molina, Santiago Villafaña, Victor García-González

**Affiliations:** 1 Facultad de Medicina y Nutrición Mexicali, Universidad Autónoma de Baja California, Humberto Torres Sanginés S/N, Centro Cívico, Mexicali 21000, BC, Mexico; andrea.munoz.ayala@uabc.edu.mx (A.M.-A.);; 2 Laboratorio Multidisciplinario de Estudios Metabólicos y Cáncer, Facultad de Medicina y Nutrición Mexicali, Universidad Autónoma de Baja California, Mexicali 21000, BC, Mexico; 3 Facultad de Odontología Mexicali, Universidad Autónoma de Baja California, Mexicali 21000, BC, Mexico; 4 Centro de Ciencias de la Salud Mexicali, Universidad Autónoma de Baja California, Mexicali 21000, BC, Mexico; 5 Centro de Innovación e Investigación en Salud, CIIS, Universidad Autónoma de Baja California, Mexicali 21000, BC, Mexico; 6 Departamento de Educación e Investigación, Servicios de Salud del Instituto Mexicano del Seguro Social para el Bienestar (IMSS-BIENESTAR), Mexicali 21000, BC, Mexico; 7 Escuela Superior de Medicina, Instituto Politécnico Nacional, Ciudad de México 11340, Mexico

**Keywords:** breast cancer, tamoxifen, chemoresistance, Nrf2, metastasis

## Abstract

Tamoxifen remains the standard treatment for estrogen receptor alpha (ER α) positive breast cancer (BC) cases. However, a significant proportion of patients develop chemoresistance, leading to disease recurrence. The Nuclear Factor Erythroid 2-Related Factor 2 (Nrf2), coded by *NFE2L2* gene, has emerged as a key player in chemoresistance and tumoral progression across multiple cancer types, including BC. This study aimed to analyze the role of Nrf2 in metastasis-related processes in a tamoxifen-metabolite-resistant BC cell variant (MCF-7^Var-H^) and to assess the impact of Nrf2 modulation. We analyzed Nrf2 expression and nuclear localization and observed that both were increased in endocrine-chemoresistant MCF-7^Var-H^ cells compared with MCF-7 parental cells. Critically, we assessed the effects of Nrf2 on migration, invasion, and metalloproteinase secretion capacity using wound-healing assays, Boyden chamber assays, and zymography, respectively. Our results suggest that Nrf2 actively promotes metastatic behaviors in the resistant variant. To further explore its pharmacological relevance, we designed and synthesized small interfering RNAs (siRNAs) targeting *NFE2L2* mRNA in its coding region by heterogeneous-phase chemical synthesis. Transfection with these siRNAs significantly inhibited metastasis-related functions such as migration in MCF-7^Var-H^ cells. Overall, siRNAs targeting Nrf2 may be promising tools for treating chemoresistant and metastatic breast cancer.

## 1. Introduction

In 2022, an estimated 2 million women were diagnosed with breast cancer (BC) globally, making it the most prevalent malignancy in this population [1]. BC tumors are classified based on estrogen receptor α (ERα), progesterone receptor (PR), and human epidermal growth factor receptor 2 (HER2) expression, determined by immunohistochemistry. ERα-positive tumors account for about 80% of all BC cases [2]. For this type of tumor in premenopausal women, tamoxifen is a highly effective treatment; however, approximately 40% percent of patients relapse due to chemoresistance acquisition [3].

In addition to chemoresistance, metastasis represents a major clinical challenge in BC. About 30% of patients with diagnosed BC develop metastasis during their lifetime [4]. Metastasis is a multistep, tightly regulated process that includes local invasion, extracellular matrix degradation, intravasation into the bloodstream, and colonization of distant organs [5,6].

To sustain metastasis-related processes and tumor progression, cancer cells must adapt to the increased metabolic demands and the associated cellular stress. In this context, the Unfolded Protein Response (UPR) is a key adaptive mechanism in multiple types of cancer, including BC [7]. The UPR is activated in response to the accumulation of misfolded proteins in the endoplasmic reticulum, a condition induced by cellular stressors. This response is coordinated by three transmembrane sensors: Inositol Requiring Enzyme 1 (IRE1), PKR-like ER Kinase (PERK), and Activating Transcription Factor 6 (ATF6) [8]. In vitro, pharmacological agents such as tunicamycin (tuni) are commonly used to induce UPR activation and study its biological effects [9,10].

Notably, the activation of the PERK signaling branch promotes Nuclear Factor Erythroid 2-Related Factor 2 (Nrf2) nuclear translocation, which is a transcription factor with pro-survival effects [11,12].

In prior research, we generated a tamoxifen-metabolite-resistant BC cell variant named MCF-7^VarH^, derived from MCF-7, an ERα-positive cell line. MCF-7^VarH^ acquired a Triple-Negative (TNBC)-like phenotype, characterized by reduced expression of hormone receptors and enhanced migratory capacity [13]. Importantly, MCF-7^VarH^ cells exhibited Nrf2 overexpression.

Nrf2 is a 605 amino acid protein with seven functional domains (Neh1–Neh7) [14]. Under physiological conditions, it modulates oxidative stress in cells by activating transcription of enzymes related to the antioxidant response, such as glutamate cysteine ligase (GCL) and glutathione peroxidase 2 (GPX2), which are crucial for glutathione synthesis, as well as heme oxygenase 1 (HO-1) and NAD(P)H quinone oxidoreductase 1 (NQO1), which are key detoxifying enzymes. However, aberrant activation of Nrf2 has been extensively implicated in cancer progression, metastasis, and chemoresistance. In addition to enhancing detoxification capacity and thereby increasing drug resistance, it promotes metastasis by increasing the expression of metalloproteases 2 and 9 (MMP-2/9) and supports cell survival by inducing the expression of anti-apoptotic proteins, including B cell leukemia/lymphoma-2 (BCL-2) and B-cell lymphoma-extra large (BCL-XL) [15].

Consistently, in breast cancer, a meta-analysis including 7096 patients demonstrated that Nrf2 overexpression correlates to reduced overall survival and disease-free survival [16]. Furthermore, Nrf2 downregulation has been shown to resensitize lung, hepatic, pancreatic, colorectal, and breast cancer cell lines to treatment, while inhibiting tumoral progression [17,18,19,20,21]. Therefore, Nrf2 represents a promising target.

While Nrf2 modulation has been explored using natural and synthetic molecules [14,22], all candidates have inherent limitations, such as poor specificity and selectivity [22]. Nevertheless, emerging biological treatments, such as small interfering RNAs (siRNAs), represent a promising alternative.

siRNAs are double-stranded, noncoding RNA molecules, typically 19–23 bases in length, which act by binding to the mRNAs with complementary sequence, preventing their translation and inducing their specific degradation. Since 2018, with the approval of Patisiran for hereditary transthyretin-mediated amyloidosis, the FDA has approved more siRNAs for the treatment of various diseases [23].

In the present work, we assessed Nrf2’s involvement in metastasis-related processes, including migration and metalloprotease 9 (MMP-9) secretion to the extracellular medium in a tamoxifen-chemoresistant BC model. We also design, synthesize, and probe Nrf2-specific siRNAs in our chemoresistant model. SiRNA transfection significantly reduced Nrf2 expression and downstream NQO1 expression. Importantly, Nrf2 downregulation also decreased migration. Results suggest that a targeted siRNA approach against Nrf2 may represent a novel and effective coadjuvant strategy for treating BC.

## 2. Results

### 2.1. UPR Activation Promotes Nrf2 Nuclear Localization in MCF-7^VarH^

**Figure 1:**
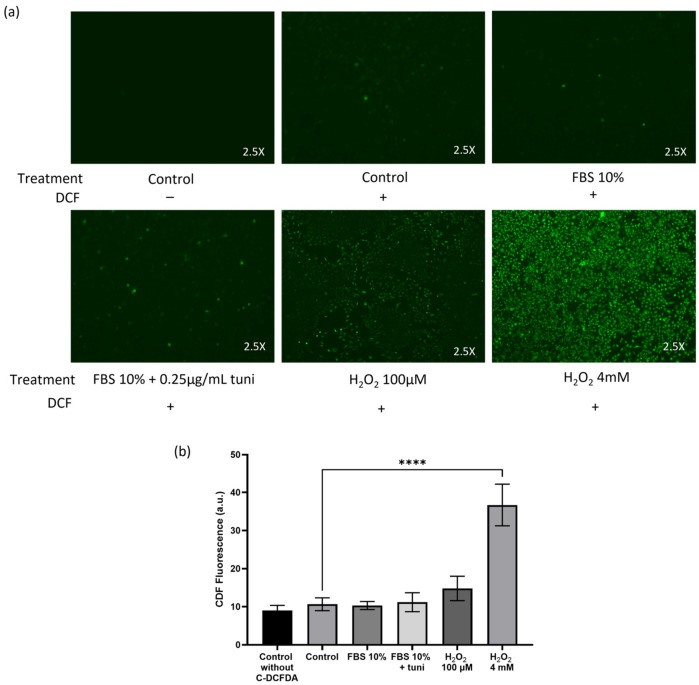
The treatment with 10% FBS plus 0.25 µg/mL Tuni did not induce significant oxidative stress in MCF-7^Var-H^. (**a**) Representative images of dichlorofluorescein (DCF) derivatives fluorescence after treatment for 24 h (channel: green). Treatments with H_2_O_2_ at 100 µM and 4 mM were included as positive controls for ROS generation (2 h). (**b**) Graph of estimated CDF fluorescence determined using ImageJ 1.53k. Mean values are presented (n = 3, mean ± SD); **** *p* < 0.0001.

Oxidative stress can activate many cellular pathways. For this reason, we chose to use a model to enhance Nrf2 activity through a mechanism independent of oxidative stress. We used Tunicamycin (tuni) at a concentration of 0.25 µg/mL to favor Nrf2 nuclear translocation and activation [10]. We observed that tuni treatment compromised cell viability, so to mitigate this adverse effect, it was administered in combination with 10% fetal bovine serum (FBS). To demonstrate that tuni treatment does not generate Reactive Oxygen Species (ROS) in a significant way, we measure dichlorofluorescein (DCF) derivatives fluorescence (492–495/517–527 nm) in MCF-7^Var-H^ chemoresistant cells treated with FBS 10%, FBS 10% + 0.25 µg/mL tuni (FBS 10% + Tuni), and experimental DMEM (control) for 24 h. DCF is formed by intracellular decarboxylation and ROS oxidation of 6-carboxy-2′,7′-dichlorodihydrofluorescein diacetate-di(acetoxymethyl ester) probe. For positive ROS generation control, we used 100 µM and 4 mM H_2_O_2_ treatments for 2 h. As shown in Figure 1a,b, FBS 10% + tuni does not result in a significant increase in DCF fluorescence compared with the control condition. In contrast, a 4 mM H_2_O_2_ treatment increased DCF fluorescence by almost 4-fold compared with the control (Figure 1b).

Later, we applied the treatments mentioned above to MCF-7 and MCF-7^Var-H^ to assess their effects on Nrf2 expression and activation.

**Figure 2:**
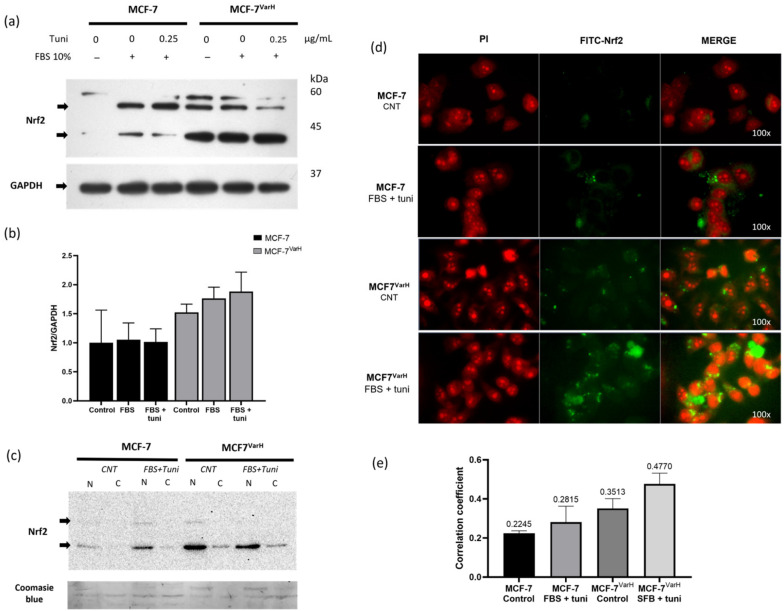
MCF-7^Var-H^ chemoresistant variant exhibits enhanced Nrf2 expression and nuclear localization. (**a**) Representative image of Nrf2 expression in MCF-7 and MCF-7^Var-H^ cells in control condition (CNT) or after FBS + tuni treatment. Arrows indicate the bands corresponding to the target. GAPDH was used as a loading control. (**b**) Graph of Nrf2/GAPDH. Mean values are presented (n = 3, mean ± SD). (**c**) Characterization of Nrf2 expression in cytoplasmic (C) and nuclear (N) fractions by Western blot in CNT and after FBS + tuni treatment. Membrane Coomassie blue staining was used as a loading control. (**d**) Epi-fluorescence images that show cell nuclei stained with propidium iodide (PI) colored in red, FITC-Nrf2 colored in green, and the merge. (**e**) Pearson’s correlation coefficient of PI and FITC; means are shown above each bar (n = 3 independent images). Control condition (CNT), FBS 10% plus tunicamycin 0.25 µg/mL (FBS + tuni).

Nrf2 expression was first assessed in MCF-7 and MCF-7^VarH^ cells using western blot (WB) analysis (Figure 2a). An overexpression of Nrf2 was observed in MCF-7^VarH^ compared with MCF-7 cells. Moreover, treatment with FBS + tuni further increased Nrf2 expression in both MCF-7^VarH^ and MCF-7 cells (Figure 2a,b).

Considering that Nrf2 exerts its activity after translocating to the nucleus, we evaluated its cellular localization under control conditions and following FBS + tuni treatment. For this purpose, we obtained nuclear and cytoplasmic fractions from both MCF-7^VarH^ and MCF-7 cells and evaluated Nrf2 abundance using WB. We found that nuclear Nrf2 was higher in MCF-7^VarH^ than in MCF-7 under control conditions. Notably, nuclear Nrf2 increased in both variants after treatment, but was more highlighted in MCF-7^VarH^ (Figure 2c). Furthermore, Nrf2 abundance in nuclear fraction isolates from both variants was contrasted directly under treated and untreated conditions (Supplementary Figure S1).

To complement the latter analysis, we determined Nrf2 localization using epifluorescence microscopy. For this assay, cells were labeled with Nrf2-FITC, and the nucleus was counterstained with propidium iodide (PI). As shown in Figure 2d, FBS + tuni treatment enhanced Nrf2 nuclear localization in MCF-7 and MCF-7^VarH^. Notably, MCF-7^VarH^ exhibited increased Nrf2-associated fluorescence and enhanced PI-FITC colocalization compared with MCF-7 under control conditions, as determined by Pearson’s correlation coefficient (Figure 2e). The FBS + tuni treatment enhanced Nrf2-associated fluorescence in both variants. Treatment also enhanced PI- FITC colocalization in both variants, with a more pronounced effect on MCF-7^VarH^ (Figure 2e). The results suggest that MCF-7^VarH^ exhibits greater Nrf2 nuclear localization than MCF-7, and that treatment with Tuni + FBS enhances Nrf2 nuclear translocation (Figure 2c–e).

Considering the previously described results, the effects of Nrf2 activation on metastasis-related processes were later evaluated in MCF-7 and MCF-7^VarH^ cells.

### 2.2. Nrf2 Activation Promotes Metastasis-Associated Processes in MCF-7^VarH^

**Figure 3:**
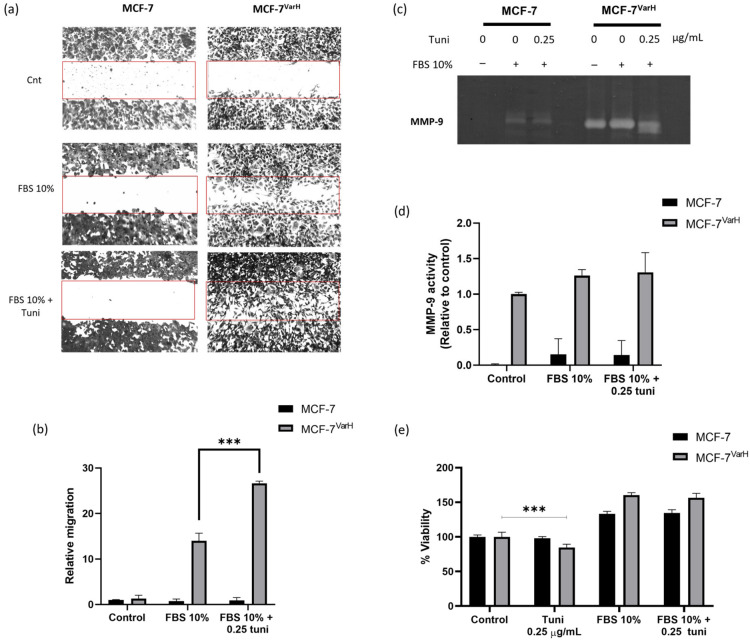
Characterization of MCF-7 and MCF-7^Var-H^ migration capacity, MMP-9 secretion, and viability after treatment. (**a**) Representative images of the wound-healing assay of MCF-7 and MCF-7^Var-H^, captured using an inverted light microscope at 4× magnification The wound area was highlighted in red. (**b**) Wound-healing assay results expressed as a percentage with respect to controls in parental and MCF-7^Var-H^ cells. Mean values are presented (n = 3, mean ± SD; *** *p* < 0.001). (**c**) Representative image of gelatin zymography showing MMP-9 enzymatic activity in the extracellular media of MCF-7 and MCF-7^Var-H^ under the described treatments. (**d**) Zymography results expressed as a percentage with respect to MCF-7 control, mean values ± SD are present. (**e**) MTT assay results are presented as the percentage of cell viability relative to the corresponding control in each cell line following treatment. Mean values are presented (n = 12, mean ± SD; *** *p* < 0.001). Control condition (Control), FBS 10% stimuli (FBS 10%), FBS 10% plus tunicamycin 0.25 µg/mL (FBS 10% + tuni).

Migration and metalloproteinase secretion were evaluated with a wound healing assay and gelatine zymography, respectively, in MCF-7 and MCF-7^Var-H^ cells (Figure 3). MCF-7 is a well-established ER+ epithelial non-invasive cell line [24], whereas, as described, MCF-7^Var-H^ exhibits TNBC-like features [13].

We observed enhanced migration to the wound site in MCF-7^Var-H^ cells following FBS plus tuni treatment compared with stimulation with FBS 10% alone (Figure 3a). Compared with the control, migration was approximately 15 times greater with 10% FBS stimulation and increased up to 25 times with the combined treatment, demonstrating a significant difference between the two conditions (*p* < 0.001) (Figure 3b). In contrast, MCF-7 cells exhibited no migration to the wound site following either FBS or FBS plus tuni stimulation (Figure 3a,b).

A zymography assay was subsequently performed to assess metalloproteinase-9 (MMP-9) activity in the extracellular medium. Increased MMP-9 activity was inferred from enhanced gelatin degradation, as evidenced by a more intense, broader clear band. In MCF-7^Var-H^, MMP-9 secretion was slightly favored by FBS plus tuni treatment compared with 10% FBS alone (Figure 3c,d). In contrast, MMP-9 activity detected in the MCF-7 extracellular medium appears to reflect the intrinsic proteolytic activity present in FBS rather than cell-derived secretion (Figure 3d).

To determine whether these effects were influenced by alterations in cell viability, we assessed the impact of the FBS plus tuni relative to FBS 10% and tuni alone in both cell variants. As shown in Figure 3e, FBS plus tuni did not compromise cell viability in either cell line. In contrast, tuni alone significantly reduced viability in MCF-7^Var-H^ cells with respect to control conditions (Figure 3e). Additionally, invasion was also evaluated using the Boyden chamber assay (Supplementary Figure S2). Invasion was observed in MCF-7^Var-H^ only when stimulated with FBS plus tuni, whereas MCF-7 showed no invasion under either condition (Supplementary Figure S2).

These results suggest that Nrf2 plays a critical role in metastasis-related processes in the MCF-7^Var-H^ chemoresistant model. Consequently, we chose to modulate Nrf2 expression using a novel biological strategy. We designed, synthesized, and evaluated small interfering RNAs (siRNAs) targeting *NFE2L2* mRNA to counteract these processes.

### 2.3. Newly Designed siRNAs Targeting NFE2L2 mRNA Effectively Reduced Its Expression and Activity in MCF-7^VarH^

Briefly, the designed siRNAs targeting *NFE2L2* mRNA, named siRNA-NRF2(UABC)-1 and siRNA-NRF2(UABC)-2, consist of 21-nucleotide sequences bearing two terminal thymine residues at the 3′ end. Both siRNAs recognize the coding sequences (CDS) of all *NFE2L2* mRNA transcript variants reported in NCBI (Section 4.9). The general characteristics of the siRNAs are summarized in Table 1.

**Figure 4:**
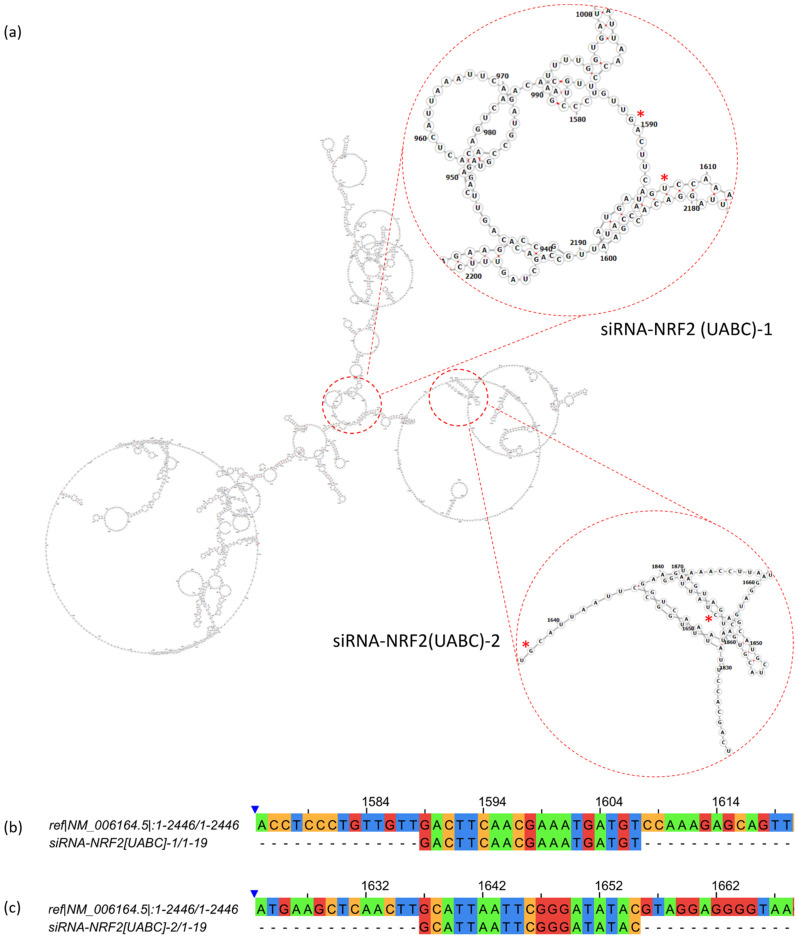
siRNA-NRF2(UABC)-1 and siRNA-NRF2(UABC)-2 interaction sites availability and selectivity evaluation by computational tools. (**a**) siRNA annealing region in centroid-predicted secondary structure of *NFE2L2* mRNA transcript variant 1 obtained in RNAfold. Red asterisks indicate the initial and terminal nucleotides of the RNA segment targeted by each siRNA. (**b**) Clustal Omega Alignment of siRNA-NRF2 (UABC)-1 with variant 1 transcript. (**c**) Clustal Omega Alignment of siRNA-NRF2 (UABC)-2 with variant 1 transcript.

siRNA-NRF2(UABC)-1 and siRNA-NRF2(UABC)-2 were selected from multiple candidates because they targeted an accessible region within the predicted structure of *NFE2L2* mRNA. They also exhibited the highest specificity and selectivity. To evaluate accessibility, we generated the predicted centroid secondary structure of *NFE2L2* mRNA variant 1 using RNAfold 2.6.0 and identified the annealing region. We found that the coordinate sites were largely unobstructed (Figure 4a). Selectivity and specificity of both siRNAs were further assessed using the NCBI BLAST tool. siRNA-NRF2 (UABC)-1 and siRNA-NRF2 (UABC)-2 targeted the CDS of all reported *NFE2L2* transcript variants with a 100% coverage, while showing no recognition of other protein-coding transcripts with more than 75% coverage. Representative alignments of siRNA-NRF2 (UABC)-1 and siRNA-NRF2 (UABC)-2 with *NFE2L2* transcript variant 1 were generated using Clustal Omega software (https://www.ebi.ac.uk/jdispatcher/msa/clustalo, accessed on 31 July 2023) and are shown in Figure 4b and Figure 4c, respectively.

As mentioned earlier, two terminal thymine residues at the 3′ end were added in both sense and antisense sequences to improve silencing efficiency, since it favors its interaction with the RISC complex [25].

**Figure 5:**
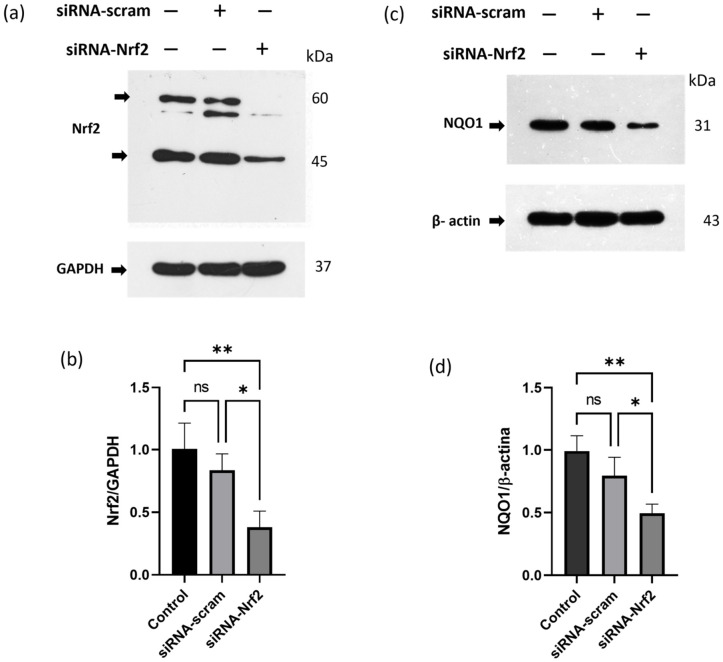
siRNA-NRF2(UABC)-1 and siRNA-NRF2(UABC)-2 transfection effectively reduced Nrf2 protein levels and NQO1 target expression in MCF-7^VarH^. (**a**) Evaluation of Nrf2 expression after Nrf2-siRNA and scramble-siRNA (siRNA-scram) transfection using Lipofectamine 3000. Arrows indicate the bands corresponding to the target. GAPDH was used as a loading control. (**b**) Graph of Nrf2/ GAPDH after transfection. Mean values are presented (n = 3, mean ± SD); ** *p* < 0.01, * *p* < 0.05, ns = non-significant. (**c**) Evaluation of NQO1 expression after Nrf2-siRNAs and scramble-siRNA (siRNA-scram) transfection using Lipofectamine 3000. Arrows indicate the bands corresponding to the target. β-actin was used as a loading control. (**d**) Graph of NQO1/β-actin after transfection. Mean values are presented (n = 3, mean ± SD), ** *p* < 0.01, * *p* < 0.05, ns = non-significant.

Later, siRNA-NRF2(UABC)-1 and siRNA-NRF2(UABC)-2 were synthesized using heterogeneous chemical synthesis, cleaved from the solid support, and purified. Finally, sense and anti-sense complementary sequences were annealed. Formation of siRNA duplex was evaluated using native polyacrylamide gel electrophoresis (Supplementary Figure S3). After evaluating siRNA concentration using a NanoDrop spectrophotometer, it was transfected into MCF-7^Var-H^ cells using Lipofectamine 3000 transfection reagent (TR). An equimolar mixture of siRNA-NRF2(UABC)-1 and siRNA-NRF2(UABC)-2 (20 pmol each) was used. This combination is hereafter referred to as siRNA-Nrf2. Nrf2 protein levels were evaluated 36 h after transfection by WB. Nrf2 expression was reduced in a statistically significant way by approximately 60% in MCF-7^Var-H^. In contrast, transfection with the siRNA-scramble did not significantly reduce Nrf2 expression (Figure 5a,b). Nrf2 target, naphthoquinone oxide reductase (NQO1), involved in the cellular antioxidant response, was also evaluated. The results show a statistically significant reduction of approximately 50% in target expression following transfection (Figure 5c,d). Scramble siRNA transfection did not significantly reduce NQO1 protein levels. Collectively, these results suggest that siRNA-Nrf2 transfection reduced Nrf2 expression and was associated with a statistically significant decrease in its activity.

To determine the effects of transfection on cell viability, we performed a clonogenic assay. We observed a reduction in colony formation following transfection. However, no statistically significant differences were detected between cells treated with the transfection reagent alone (Lipofectamine 3000) and cells transfected with siRNA-Nrf2. These findings suggest that the decrease in colony formation was primarily due to Lipofectamine’s effect on cell viability (Supplementary Figure S4a,b).

**Figure 6:**
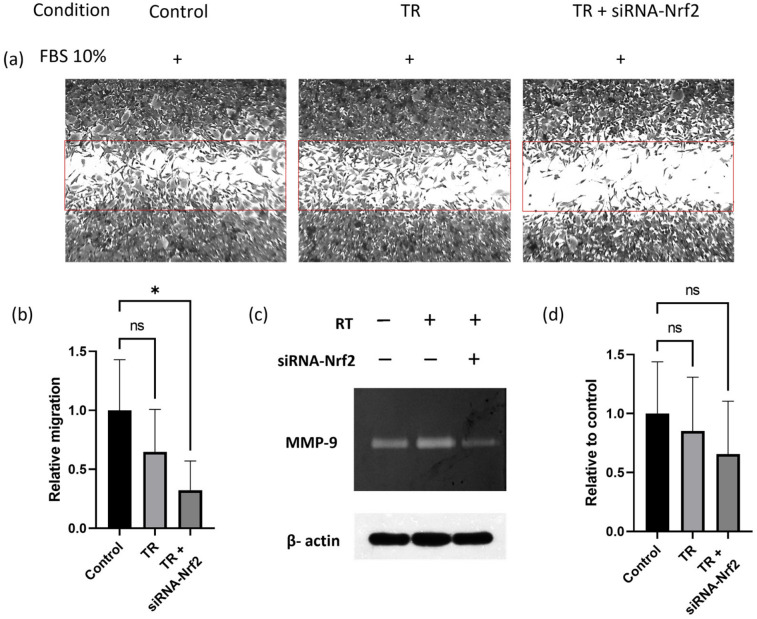
Nrf2 siRNA transfection effectively reduces metastasis-related processes in MCF-7^VarH^. (**a**) Representative microphotographs of Nrf2 MCF-7^VarH^ wound healing assay in non-transfected (control), lipofectamine 3000-treated (transfection reagent, TR), and siRNA-Nrf2-transfected cells (TR+ siRNA-Nrf2), all conditions were stimulated with 10% FBS. Images were captured using an inverted light microscope at 4× magnification. The wound area was highlighted in red. (**b**) Graph of MCF-7^VarH^ migration in the evaluated conditions relative to the control. (**c**) Representative image of MMP-9 activity in extracellular media after transfection. (**d**) Graph of MMP-9 secretion after transfection relative to control. Mean values are presented (n = 3, mean ± SD), * *p* < 0.05, ns = non-significant. TR = Transfection reagent.

Finally, metastasis-related processes, such as migration and metalloproteinase secretion, were evaluated in MCF-7^Var-H^ cells after transfection. As shown in Figure 6a,b, cell migration to the wound site was statistically significantly reduced by siRNA-Nrf2 transfection compared with treatment with the transfection reagent alone. In addition, MCF-7^Var-H^ extracellular MMP-9 secretion was reduced in siRNA-Nrf2-transfected cells, although this reduction was not statistically significant (Figure 6c,d).

Collectively, these results suggest that Nrf2 knockdown can reduce metastatic processes without significantly affecting cell viability. Therefore, modulation of Nrf2 using siRNA-based strategies may represent a novel approach for chemoresistant and/or metastatic breast cancer, potentially as a coadjuvant treatment. However, the scope of these findings is limited, and further preclinical and clinical investigations are required.

## 3. Discussion

Tamoxifen remains the preferred adjuvant treatment for patients with ERα+ tumors. However, despite its clinical efficacy, up to one-third of patients experience relapse within fifteen years of treatment initiation [26]. This is largely attributed to the acquisition of chemoresistance and the development of metastasis. Therefore, identifying novel adjuvant therapeutic strategies to counteract chemoresistance and limit metastatic development is critical.

We previously reported the generation of several tamoxifen-metabolite-resistant variants [13,27]. Specifically, a cell variant, MCF-7^VarH^, derived from the MCF-7 cell line, exhibited enhanced migration and increased secretion of matrix metalloproteases compared with the parental MCF-7 cells. Notably, MCF-7^VarH^ displayed Nrf2 overexpression relative to parental cells (Figure 2a). Consistent with our findings, extensive prior literature has linked Nrf2 overexpression and overactivation with chemoresistance [28], tumor progression, and poor clinical outcomes in several cancer types, including BC [29].

To further explore Nrf2’s role in our cellular chemoresistance model, we induced its translocation and activity using tunicamycin plus FBS 10%. Since tunicamycin activates Nrf2 via mechanisms independent of oxidative stress in other cancer models [10], we corroborated this in our system by characterizing ROS generation (Figure 1). After treatment, we observed increased Nrf2 expression and nuclear localization, particularly in MCF-7^VarH^ cells.

We subsequently evaluated the implications of Nrf2 overactivation on metastasis-related processes. Upon treatment with tuni + FBS, MCF-7^VarH^ cells exhibited increased migratory and invasive capabilities, as well as enhanced secretion of matrix metalloproteinases. In contrast, MCF-7 cells did not display migration, invasion, or metalloproteinase secretion in response to either FBS or the combination of FBS plus tuni, as expected for a low-invasive cell phenotype [24] (Figure 2). The mechanisms underlying Nrf2 upregulation of migration capacity and metalloproteinase secretion have been partially described in the literature. The promoter region of the MMP-9 gene contains two functional ARE sequences, which can mediate MMP-9 transcription in response to Nrf2 signaling [30]. In a clinical context, using the Kaplan-Meier plotter [31], we identified that high expression of *NFE2L2*, *NQO1*, and *MMP-9* mRNAs is correlated with lower survival probability in Tamoxifen-treated patients (Supplementary Figure S5). These findings further support the involvement of Nrf2 overactivation in promoting a more aggressive phenotype in tamoxifen-resistant MCF-7^VarH^ breast cancer cells.

Based on these observations, we proceeded to modulate Nrf2 expression using newly designed siRNAs targeting *NFE2L2* mRNA. Although several Nrf2 inhibitors have been identified [14], limitations in their specificity and safety profiles have prevented their advancement in clinical studies [32]. In contrast, siRNA-based approaches address selectivity concerns by binding to their targets, thereby minimizing off-target effects. Additionally, siRNA production is generally more cost-effective compared with other biological strategies, such as monoclonal antibody treatments [33].

Since the FDA approval of Patisiran (Alnylam Pharmaceuticals, Inc.) in 2018 for the treatment of transthyretin-mediated amyloidosis, multiple siRNAs have been approved for a wide range of diseases [34]. In the cancer context, siRNAs targeting the androgen receptor and the ephrin type-A receptor, as well as the Kras G12D mutation, are currently undergoing phase I clinical trials for advanced cancers and pancreatic cancer, respectively [34]. Moreover, a siRNA targeting microRNA-92 is currently being evaluated in phase I clinical trials for hematological malignancies [34].

Despite the availability of numerous siRNAs targeting Nrf2 for research applications, none have yet been evaluated in clinical trials as anticancer therapy [35]. Given the well-established role of Nrf2 in cancer progression and therapeutic resistance, siRNA-based strategies targeting Nrf2 downregulation are a promising therapeutic approach.

For the siRNA-NRF2(UABC)-1 and siRNA-NRF2(UABC)-2 obtention, multiple 19-nucleotide candidate sequences were generated using the siRNA Wizard tool by introducing the CDS of *NFE2L2* transcript variant 1. Candidate selection was guided by analyses of target site accessibility, specificity, and selectivity using RNAfold 2.6.0 (http://rna.tbi.univie.ac.at/cgi-bin/RNAWebSuite/RNAfold.cgi, accessed on 31 July 2023), NCBI BLAST 2.13.0, and Clustal Omega software (https://www.ebi.ac.uk/jdispatcher/msa/clustalo, accessed on 31 July 2023), respectively (Figure 4). The most promising sequences were chemically synthesized, purified, and annealed to produce double-stranded siRNAs.

We used an equimolar siRNA pool (siRNA-NRF2(UABC)-1 /siRNA-NRF2(UABC)-2) to maximize target silencing while limiting potential off-target activity [36]. Transfection resulted in a significant reduction in Nrf2 expression (approximately 60%), and a concomitant decrease in the expression of the downstream target gene NQO1 (approximately 50%) in MCF-7^VarH^ (Figure 5). siRNA-mediated Nrf2 knockdown did not affect colony-forming capacity (Figure S3). Although Nrf2 is not essential for cell survival, it has been associated with increased proliferation and resistance to apoptosis in tumor cells [13] and in chemoresistant cells [28]. Nrf2 downregulation achieved in this study may not have been sufficient to impair proliferative capacity, suggesting that additional signaling pathways help maintain cell proliferation in this endocrine-resistant cellular model.

In parallel, metastasis-related processes were evaluated following Nrf2 downregulation in our chemoresistant model. siRNA-mediated knockdown resulted in a significant reduction in migration toward the wound site, indicating attenuation of metastatic potential. Metalloproteinase secretion was also reduced. While Nrf2 overactivation has been strongly linked to metastasis-related processes [15], it is important to recognize that metastasis is regulated by multiple interconnected pathways. In this study, only a single molecular target was modulated; thus, combinatorial approaches using multiple siRNAs may be more effective.

Although the effects of siRNA-Nrf2 transfection in MF-7^VarH^ cells appear promising, further studies are required to evaluate the efficacy of this approach in additional breast cancer cell lines and also evaluate its safety in more complex biological models. Such validation is essential to determine its potential utility as a coadjuvant therapeutic strategy.

One of the major challenges associated with in vivo siRNA applications is their limited stability in biological environments, which constrains efficient delivery to target tissues. To address this, chemical modifications, such as 2′-methoxy (2′-OMe) or 2′-fluoro (2′-F) group substitution on the siRNA ribose, are commonly employed to enhance nuclease resistance and improve pharmacokinetic properties [33]. Building on this approach, future studies incorporating chemically modified nucleotides may provide valuable insights into improving silencing efficiency. Beyond stability, poor cellular uptake represents another significant limitation. In response, various viral and non-viral delivery systems have been developed to enhance siRNA delivery [33]. Among non-viral strategies, lipid-based delivery systems and siRNA conjugates have shown considerable promise. For example, siRNAs can be conjugated to peptides, aptamers, antibodies, or high-density lipoprotein (HDL) particles [33]. Notably, all FDA-approved siRNA therapeutics to date are N-acetyl-D-galactosamine (GalNAc) conjugates [34]. Taken together, from a translational perspective, developing an effective, targeted delivery system will be a critical step toward the clinical application of Nrf2-directed siRNA therapies.

## 4. Materials and Methods

### 4.1. Materials

Tissue culture plates were obtained from Corning (Durham, NC, USA), and cell culture reagents were acquired from Thermo Fisher (Rockford, IL, USA). Solid tunicamycin was acquired from Sigma-Aldrich (St. Louis, MO, USA). Monoclonal antibodies anti-NRF2, anti-H3 histone, and anti-GAPDH were obtained from Santa Cruz Biotechnology (Dallas, TX, USA). Anti-mouse secondary antibodies were purchased from Thermo Fisher (Rockford, IL, USA). Immobilon Kit from Millipore (Burlington, MA, USA) was used for chemiluminescence detection. Lipofectamine 3000 was acquired from Invitrogen (Carlsbad, CA, USA).

### 4.2. Cell Culture

MCF-7 cells were obtained from American Type Culture Collection (ATCC, Manassas, VA, USA), MCF-7^VarH^ cells were generated previously in our laboratory [13]. Both were conserved in Dulbecco’s modified Eagle Medium (DMEM), supplemented with 10% fetal bovine serum, 50 U/mL penicillin, 50 μg/mL streptomycin, and insulin.

### 4.3. Reactive Oxygen Species (ROS) Evaluation

Cells were seeded onto coverslips placed in 20 mm plates at a density of 400,000 cells/plate and allowed to adhere overnight. Cells were fasted for 2 h, and then the treatments were added (Control, FBS 10%, FBS 20% + tuni 0.25 µg/mL). A total of 100 µM and 4 mM H_2_O_2_ were added as positive controls and incubated for only 2 h. Later, cells were incubated with 10 uM of 6-carboxy-2′,7′-dichlorodihydrofluorescein diacetate-di(acetoxymethyl ester) (C-DCFDA, Invitrogen, Molecular Probes, Eugene, OR, USA) for 45 min at 37 °C, as described previously [37]. Finally, the dichlorofluorescein (DCF) derivatives fluorescence signals (492–495/517–527 nm) generated by cellular esterases and ROS oxidation were documented using the cell counter Countess II FL, EVOS Light Cube GFP 2.0.

### 4.4. Fluorescence Microscopy

Cells were seeded onto coverslips in an untreated 6-well dish at a density of 400,000 cells/well and allowed to proliferate until reaching 60–70% confluence. Cells were fasted for 2 h, then treated. Later, cells were washed with PBS, fixed with 2% paraformaldehyde/PBS, permeabilized with 0.01% Triton X-100, and blocked with 2% bovine serum albumin/PBS. Cells were incubated with anti-Nrf2 primary antibody, with FITC-coupled secondary antibody, and counterstained with Propidium Iodide. Finally, cells were observed under an EPI-fluorescence microscope (Axio VertA.1, Zeiss, Gottingen, Germany) as described in previous work [38]. Pearson correlation coefficients for fluorescence microscopy images were calculated using ImageJ 1.53k.

### 4.5. Nuclei Isolation

Cells were seeded in 6-well dishes (400,000 cells/well) and allowed to proliferate until reaching 90–95% confluency. Cells were fasted for 2 h, and then the treatment was added. Later, cells were washed with PBS, 500 µL of new PBS was added and cells were scraped. Cells in suspension were recovered in an Eppendorf and centrifuged at 2000× *g* rpm for 2 min at 20 °C. Supernatant was removed and the cells were resuspended in 400 µL of Sucrose (250 mM)/Imidazole (3 mM) buffer, supplemented with protease and phosphatase inhibitors. Cell suspension was passed through a 30 G syringe 35 times. To verify nuclear isolation and integrity, we used Trypan Blue Staining. Nuclei were recovered by centrifugation at 3400 rpm for 15 min. After the centrifugation, the supernatant was collected and identified as the cytoplasm. The pellet was identified as the nucleus. Both fractions were lysed with a lysis buffer and analysed by Western blot. This protocol was adapted from previous works [37].

### 4.6. Cellular Lysis and Western Blot

Cells were seeded in 6-well dishes (400,000 cells/well) and allowed to proliferate until reaching 90–95% confluency. Cells were fasted for 2 h, and treatments were added. After treatment, cells were washed with 1X PBS and lysed with a lysis buffer supplemented with protease and phosphatase inhibitors to obtain a protein extract. Samples (25 μg/lane) were run in SDS-PAGE gels and subsequently transferred to PVDF membranes. Membranes were blocked with TBS/0.1% Tween-20 and 5% low-fat milk for 1 h at 37 °C. Next, membranes were incubated with the corresponding primary antibodies at 4◦C overnight, followed by incubation with horseradish peroxidase-conjugated (HRP) secondary antibodies for 120 min at 37 °C, as described in our previous methodology [39]. HRP activity was analyzed with Immobilon Western Kit from Millipore. Band intensities were measured using ImageJ 1.53k.

### 4.7. Clonogenic Assay

Cells were seeded in 20 mm dishes at a density of 5000 cells per dish. After 24 h of adhesion, cells were treated with DMEM supplemented with 10% FBS. Cultures were maintained for 4 days, fixed with 4% paraformaldehyde, and stained with Coomassie Blue. This methodology was adapted from a described protocol [40].

### 4.8. Migration Assays

For the wound-healing assay, cells were seeded in 6-well dishes (400,000 cells/well) and allowed to proliferate to 95–100% confluency. Cells were treated with Mitomycin C for 2 h to prevent proliferation. Scratches were made using a micropipette tip, and treatments were applied. Finally, cells were fixed and stained with Coomassie blue. Adapted from [41].

### 4.9. Zymography

Cells were seeded in 6-well dishes (400,000 cells/well) and allowed to proliferate until reaching 90–95% confluency. Treatments were added, and the extracellular medium was collected. Extracellular medium was centrifuged at 8000× *g* rpm for 10 min. Samples were prepared in non-denaturing 5× sample buffer and loaded onto 8% polyacrylamide gels copolymerized with 1% gelatin, then run in an electrophoresis chamber. Later, gels were washed with a 2.5% Triton X-100 solution and incubated in development buffer for 72 h at 37 °C. Gels were stained with 0.25% Coomassie Blue G-250 in 10% acetic acid and 30% methanol, as described in our previous work [42].

### 4.10. Cell Viability Assay

Cells were seeded in 96-well plates (20,000 cells/well) and allowed to proliferate until reaching 75% confluency. DMEM-supplemented medium was replaced with Opti-MEM and incubated at 37 °C for 2 h. Later, treatments were added and incubated for 24 h. After treatment time, 30 µL of dimethylthiazol-2-yl-2,5-diphenyltetrazolium bromide (MTT, Sigma-Aldrich, St. Louis, MO, USA) at 2.1 g/mL was added and incubated at 37 °C for 4 h. Formazan crystals generated by mitochondrial cell activity were dissolved with a lysis buffer (20% SDS, 50% N,N-dimethyl-formamide, pH 3.7). Finally, absorbances were determined at 590 nm after 12 h.

### 4.11. SiRNAs Design and Synthesis

Several siRNA sequences were obtained with Wizard (InvivoGen) based on the coding region of the *NFE2L2* mRNA transcripts reported in the NCBI database (Transcript variant 1: NM_006164.5, transcript variant 2: NM_001145412.3, transcript variant 3: NM_001145413.3, transcript variant 4: NM_001313900.1, transcript variant 5: NM_001313901.1, transcript variant 6: NM_001313902.2, transcript variant 7: NM_001313903.2, transcript variant 8: NM_001313904.1). The candidate sequences were then evaluated for specificity, selectivity, and predicted interaction sites using the NCBI BLAST tool and RNAfold 2.6.0 (http://rna.tbi.univie.ac.at/cgi-bin/RNAWebSuite/RNAfold.cgi, accessed on 31 July 2023). The alignment of siRNA sequences with transcript variant 1 was analyzed using Clustal Omega [43] and visualized using Jalview 2.11.5.1. The two sequences showing the most favorable parameters were selected for synthesis. Sense and antisense strands of each siRNA were synthesized using a Mermade-8 BioAutomation system through standard solid-phase chemical synthesis. Following synthesis, oligonucleotides were cleaved from the solid support with an ammonium hydroxide (NH_4_OH) solution, purified using alcohol precipitation, and quantified spectrophotometrically at 260 nm. The sense and antisense strands were annealed to generate double-stranded siRNAs. Finally, siRNA concentration was determined prior to transfection using the NanoDrop 1000 spectrophotometer (Thermo Fisher, Wilmington, DE, USA).

### 4.12. Native Polyacrylamide RNA Electrophoresis

A total of 20 μg of each siRNA was loaded onto a native polyacrylamide gel and electrophoresed in TAE (Tris–acetate–EDTA) buffer at 90 V. The gel was visualized by silver staining. Briefly, the gel was first fixed in a solution of ethanol and glacial acetic acid for 1 min, then incubated in a silver nitrate staining solution for 10 min. Later, the gel was transferred to a freshly prepared developing solution (sodium hydroxide/ formaldehyde) and finally maintained in a sodium carbonate storage solution. The gel was documented using a UV transilluminator.

### 4.13. siRNAs Transfection

Cells were seeded in 12-well dishes (200,000 cells/well) and allowed to proliferate until reaching 40–60% confluency. A total of 20 pmol of siRNA-NRF2(UABC)-1 and siRNA-NRF2(UABC)-2, and 4.5 μL of Lipofectamine 3000 were used per well, and the mixture was incubated for 24 h. Cells were maintained in complete DMEM for another 12 h, then the cells were fasted, and treatments were added. Finally, supernatant media was collected, and cell lysis and protein extraction were carried out. As a transfection control, the same protocol was used, except that 40 pmol of siRNA-Nrf2 was replaced with an equal amount of siRNA-scramble. This protocol was adapted from the manufacturer’s recommendations.

### 4.14. Statistical Analysis

Data are presented as mean ± SD. Statistical analyses were performed using GraphPad Prism software version 9.4.1. One-way ANOVA followed by Dunnett’s multiple comparisons post hoc test was used to assess statistical significance. A statistically significant result is defined by a *p*-value < 0.05.

## 5. Conclusions

We have shown that the Nrf2 pathway plays a significant role in metastasis-associated processes in the endocrine-resistant MCF-7^VarH^ cells. Nrf2 activation, independent of oxidative stress, favored processes such as cell migration and MMP-9 secretion in MCF-7^VarH^ cells. To downregulate the Nrf2 pathway, we designed and synthesized two Nrf2-targeting siRNAs and successfully transfected MCF-7^VarH^ cells with them. Notably, following siRNA transfection, we observed a significant decrease in cell migration and in MMP-9 secretion, both of which were dependent on Nrf2 expression. Although several Nrf2 modulators are currently available, none have yet met the required safety standards to be considered a viable treatment. The siRNAs developed in this study may offer a potential novel therapeutic approach for managing complex breast cancer cases. However, further in vitro and in vivo studies are needed to fully evaluate their therapeutic potential.

## Figures and Tables

**Table 1:** Description of the designed Nrf2 siRNAs’ general characteristics.

	siRNA-NRF2(UABC)-1	siRNA-NRF2(UABC)-2
Sense	5′-GACTTCAACGAAATGATGTTT-3′	5′-GCATTAATTCGGGATATACTT-3′
Antisense	3′-TTCTGAAGTTGCTTTACTACA-5′	3′-TTCGTAATTAAGCCCTATATG-5′
% GC	36.84%	38.10%
MW (g/mol)	12,830	12,830.4
Annealing region	CDS	CDS
Coordinate	1589–1607	1637–1655

% GC—% of guanines and cytosines, MW—molecular weight, CDS—Coding sequence.

## Data Availability

The original contributions presented in this study are included in the article/Supplementary Material. Further inquiries can be directed to the corresponding author.

## Supplementary Materials

The following supporting information can be downloaded at: https://www.mdpi.com/article/10.3390/ijms27104506/s1.

## Abbreviations

The following abbreviations are used in this manuscript:

ERα+	Estrogen receptor alpha-positive
BC	Breast cancer
Nrf2	Nuclear Factor Erythroid 2-Related Factor 2
siRNA	small interfering RNA
PR	Progesterone receptor
HER2	Human epidermal growth factor receptor 2
EMT	Epithelial–mesenchymal transition
TNBC	Triple-Negative Breast Cancer
GCL	Glutamate cysteine ligase
GPX2	Glutathione peroxidase 2
HO-1	Heme oxygenase 1
NQO1	NAD(P)H quinone oxidoreductase 1
MMP-9	Metalloproteases 9
tuni	tunicamycin
CDS	Coding region
