# Supplementary material for: Nrf2 Is Associated with Metastasis-Related Processes in a Chemoresistant Breast Cancer Model: Insights from siRNA Modulation
Source: Int J Mol Sci. 2026 May 18;27(10):4506. doi: 10.3390/ijms27104506 (PMC13207780; doi:10.3390/ijms27104506)
Supplement: Supplementary file 1 [file ijms-27-04506-s001.zip › ijms-4169430-supplementary.pdf]

Supplementary figures

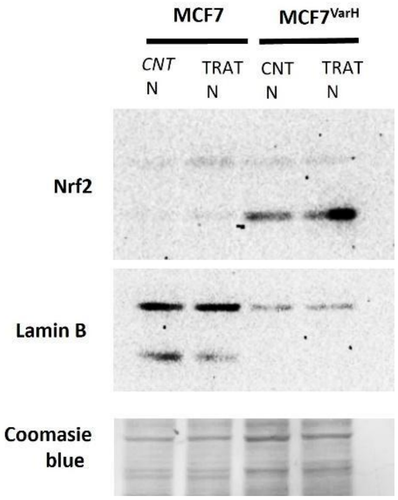

Figure S1. Nrf2 abundance in nuclear isolates and total lysates of MCF-7^{Var-H} and MCF-7. Nuclear Nrf2 was contrasted in control condition and after treatment in both variants. Nrf2 expression was also evaluated in total lysates of MCF-7^{Var-H} and MCF-7. Lamine B was used as nuclear loading control and the Coomasie blue staining of the membranes was used as a general loading control.

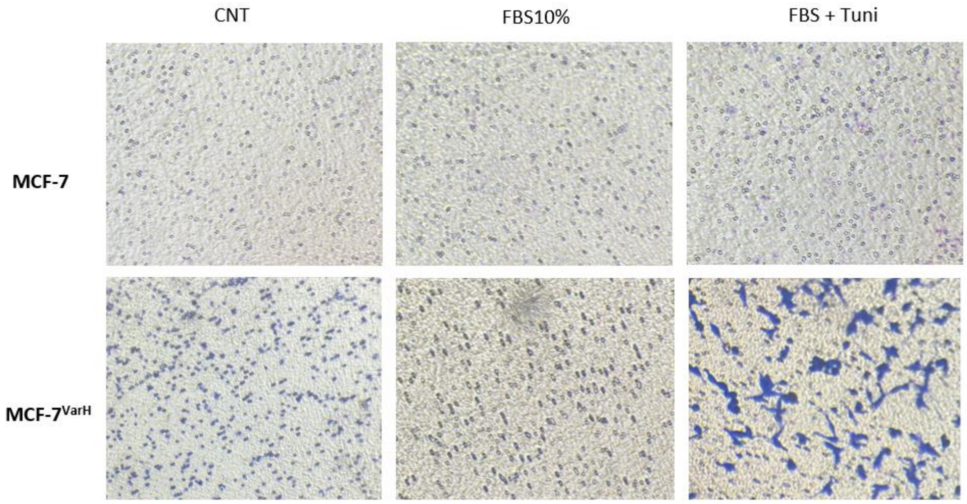

Figure S2. Evaluation of MCF-7 and MCF-7^{Var-H} cell invasion after Nrf2 activation by Boyden chamber assay. Representative image invasion assay of MCF-7 and MCF-7^{Var-H} cells under CNT, FBS 10%, and FBS+tuni treatments. Invasive cells were fixed and stained with Coomasie blue.

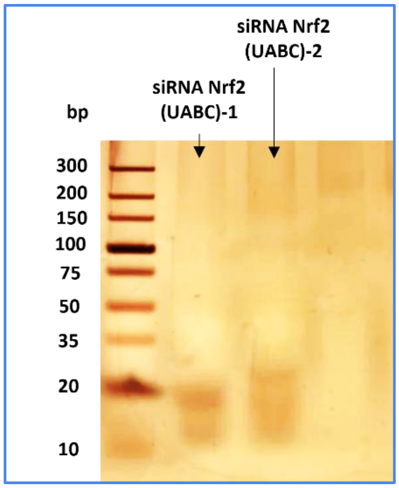

Figure S3. SiRNA Nrf2 (UABC)-1 and SiRNA Nrf2 (UABC)-2 polyacrylamide gel electrophoresis. The gel was revealed using silver staining.

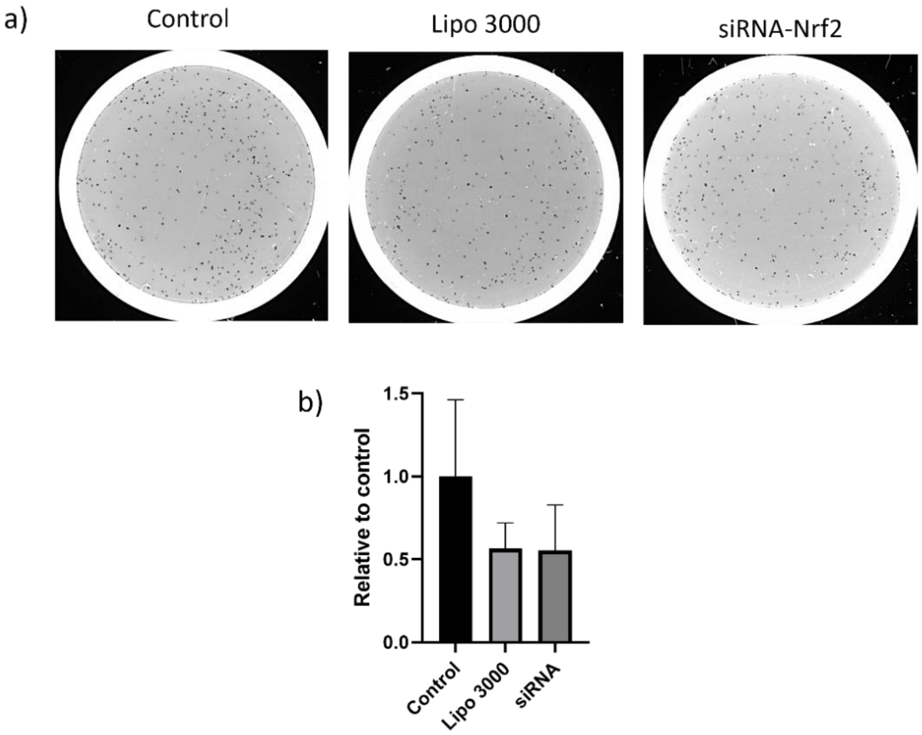

Figure S4. MCF-7^{VarH} clonogenic assay after siRNA-Nrf2 transfection. a) Representative photographs of MCF-7^{VarH} after clonogenic assay. b) Graph of MCF-7^{VarH} colony formation after transfection relative to control (n = 3, mean ± SD). Lipofectamine 3 000 (Lipo 3000).

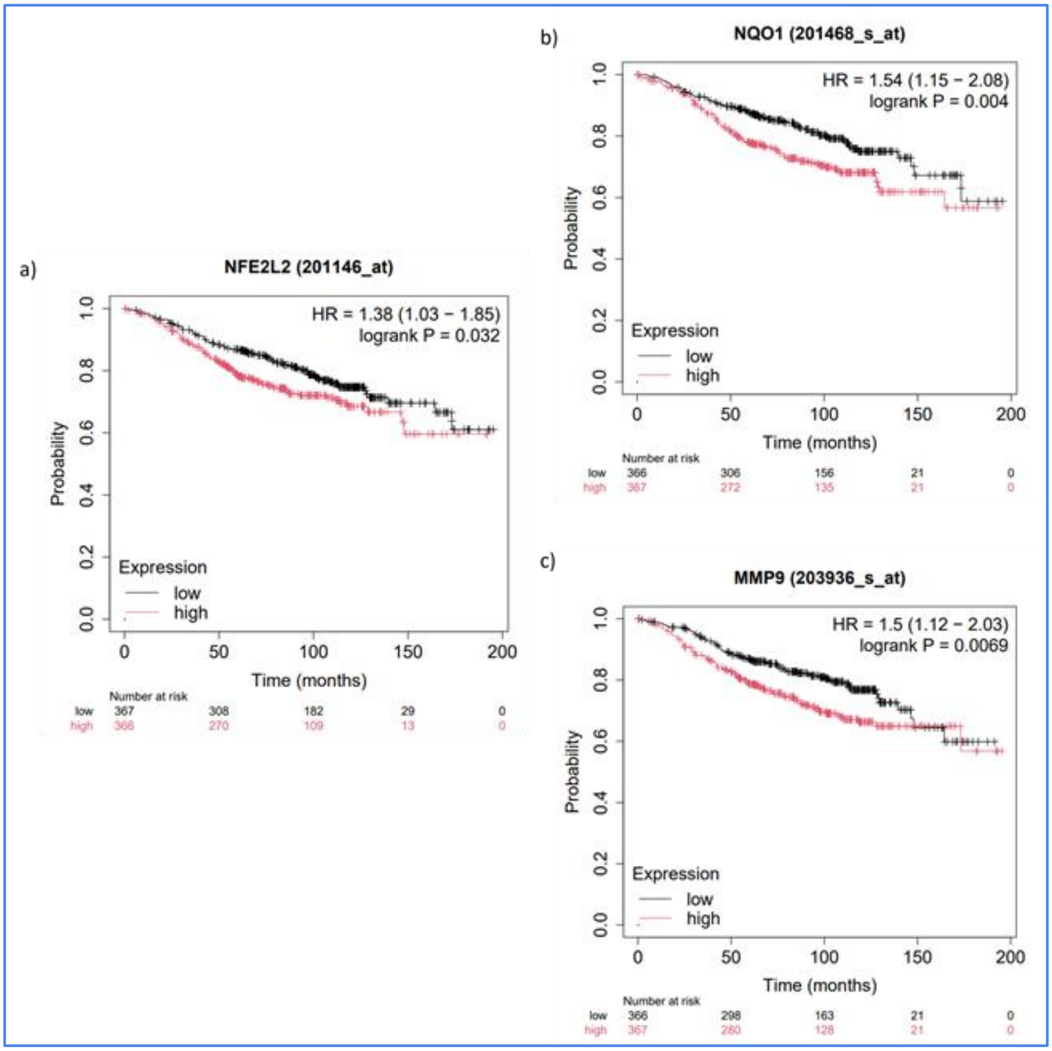

Figure S5. Kaplan-Meier survival analysis of the Nrf2 axis in tamoxifen-treated patients. a) Kaplan-Meier curve showing the association between NFE2L2 expression and patient survival. b) Kaplan-Meier curve showing the association between NQO1 expression and patient survival. c) Kaplan-Meier curve showing the association between MMP9 expression and patient survival.
